# How Doctors Are Made

**Published:** 1891-09-19

**Authors:** 


### EDITOR'S LETTER-BOX.

[Our correspondents are reminded that prolixity is a great bar to publication, and that brevity of style and conciseness of statement greatly facilitate early insertion].

### "HOW DOCTORS ARE MADE."

Sir,—In your article on "How Doctors are Made," you make the remark that in the case of a student taking the London degree "he will not have a University education, but only a hospital education." May I protest against this? At the provincial college I have the honour to be a student of, viz., Owens, we possess a faculty of Arts, Science, and Law, just as efficient as that of Edinburgh, and in many cases the medical student of Owens graduates either as B.A. or B.Sc. (chiefly the latter) before proceeding with his purely medical work.

In this case, why do we not get as efficient a University education as at Cambridge?

It seems to me that in your article you have overlooked one of the most important points which ought to have been considered, viz., Where does your correspondent live? If he lives near a good provincial college, where his son could attend during the day, return home for the evenings, let him, by all means, send his son there. He will in this way have full control over his son's actions, and will also greatly lessen the expense.

If, on the other hand, he does not live sufficiently near a college for this plan to be feasible, there are in some cases, *e.g.*, Owens, resident colleges officially attached to the day college, where the students are under the supervision of the college authorities, and also have special tutors of their own besides.

To the uninformed there is, I admit, a prejudice in favour of one of the older Universities, but this is being rapidly overcome, and the London degree is now beginning to be looked upon as the "blue ribbon" of the medical profession.—I am, yours very sincerely, B.Sc. (Lond.).

Owens College, Manchester,
September 11th, 1891.

